# Concour for Demonstrator of Anatomy in Rush Medical College

**Published:** 1850-07

**Authors:** 


### ARTICLE IV.

### CONCOUR FOR DEMONSTRATOR OF ANATOMY IN RUSH MEDICAL COLLEGE.

The concour for the place of Demonstrator of Anatomy in Rush Medical College, came off on the 17th and 18th of June last. Only two competitors came forward, out of a number who had given the required notice. They were Dr. J. W. Freer, of Wheeling, and Dr. E. S. Cooper, of Peoria, Illinois.

The trial was highly creditable to both, and resulted in the appointment of Dr. Freer.

The preparations presented by Dr. Freer, for the inspection of the Faculty, all of which have been made by him since the announcement of the concour, last winter, were very numerous, and would compare favorably with those from the hand of any other anatomist in any country.

This first trial of the concour system on this side of the Atlantic, has satisfied all concerned of its superiority over any other plan for selecting teachers ; and although there may be circumstances where it would be inexpedient, for want of time or other necessary condition, still we feel confident that its general adoption will be found of the utmost utility, both to institutions and the profession at large. E.

